# Automating a Dehazing System by Self-Calibrating on Haze Conditions

**DOI:** 10.3390/s21196373

**Published:** 2021-09-24

**Authors:** Dat Ngo, Seungmin Lee, Gi-Dong Lee, Bongsoon Kang

**Affiliations:** Department of Electronics Engineering, Dong-A University, Busan 49315, Korea; datngo@donga.ac.kr (D.N.); 1672885@donga.ac.kr (S.L.); gdlee@dau.ac.kr (G.-D.L.)

**Keywords:** automation, self-calibration, dehazing, haze density, image blending, multiscale fusion

## Abstract

Existing image dehazing algorithms typically rely on a two-stage procedure. The medium transmittance and lightness are estimated in the first stage, and the scene radiance is recovered in the second by applying the simplified Koschmieder model. However, this type of unconstrained dehazing is only applicable to hazy images, and leads to untoward artifacts in haze-free images. Moreover, no algorithm that can automatically detect the haze density and perform dehazing on an arbitrary image has been reported in the literature to date. Therefore, this paper presents an automated dehazing system capable of producing satisfactory results regardless of the presence of haze. In the proposed system, the input image simultaneously undergoes multiscale fusion-based dehazing and haze-density-estimating processes. A subsequent image blending step then judiciously combines the dehazed result with the original input based on the estimated haze density. Finally, tone remapping post-processes the blended result to satisfactorily restore the scene radiance quality. The self-calibration capability on haze conditions lies in using haze density estimate to jointly guide image blending and tone remapping processes. We performed extensive experiments to demonstrate the superiority of the proposed system over state-of-the-art benchmark methods.

## 1. Introduction

Outdoor imaging is subject to environmental effects, such as lighting and weather conditions. Therefore, captured images occasionally exhibit inconvenient characteristics (for example, faint color, contrast reduction, and loss of details), posing practical difficulties for image processing algorithms deployed in high-level vision applications. In real-world scenarios, light scattering and diffusion in the turbid atmosphere are probably the most common causes of image degradation. Researchers widely refer to these degradation sources as haze, which comprises microscopic aerosols occurring naturally or originating from industrial emissions. Recently, Pei et al. [1] investigated the effects of image degradation on object recognition and discovered that the accuracy decreased with increasing haze. This discovery, coupled with the sheer impracticality of improving existing algorithms to reverse the image degradation, requires image dehazing as a pre-processing step for visibility restoration. Since then, owing to its promising potential in consumer photography and computer vision applications, image dehazing has garnered significant importance, attracting unceasing scientific attention over the previous decades. The current effort-intensive trend towards development of autonomous vehicles (AVs) is a prime example. For replacing the human driver, AVs must be equipped with state-of-the-art visual sensors with all-weather reliability. However, this task is still unattainable because the elements limit the functionality of even the most advanced sensors, rendering image dehazing very relevant for overcoming this inevitable difficulty. Furthermore, according to The United States Department of Transportation [2], nearly 22 percent of vehicle crashes occurring each year are weather-related, which calls for groundbreaking research to facilitate AV operations in adverse weather [3].

Diverse algorithms ranging from simple histogram equalization to complex deep neural networks have been proposed to address the limited visibility in hazy weather. Among the existing methods, those based on optical physics are perhaps the most commonly encountered in the literature. However, they are dependent on certain assumptions about the transmission medium to alleviate the ill-posed problem of image dehazing. Consequently, the dehazing performance may suffer in case the imposed assumptions fail. For example, the well-publicized dark channel prior (DCP) proposed by He et al. [4] assumes that local image patches contain very dark pixels with approximately zero intensity in at least one color channel. Notably, the DCP remains valid as long as the image does not contain sky regions or shady objects. In the former condition, the presence of high-intensity bright pixels in all color channels causes the breakdown of the DCP, whereas in the latter, shadow conceals the actual pixel values, affecting its accuracy. Furthermore, virtually all existing dehazing algorithms assume a homogeneous atmosphere, with constant atmospheric light over the entire image. Although this assumption simplifies the lightness estimation, it may result in post-dehazing artifacts in a heterogeneous atmosphere. For example, hazy nighttime images typically contain several light-emitting sources, such as streetlamps and billboards. Their effects on the imaging process must be considered for accurate visibility restoration.

Another school of thought attempted to address the aforementioned issue by approaching image dehazing from the image enhancement perspective. In this context, researchers focused on enhancing the fundamental aspects of perceptual image quality such as contrast, sharpness, and colorfulness. One of the earliest attempts in this field involved the exploitation of traditional image-processing techniques. Kim et al. [5] adopted block-overlapped histogram equalization to enhance the image contrast, increasing the image visibility significantly. Despite such an impressive contrast enhancement, this method leaves the haze unaffected because it does not consider the hazy image formation. Subsequent research leveraged image fusion, which combines several images to produce a single image that inherits the desirable characteristics from the source images. Ancuti et al. [6,7,8], in a series of important studies in this field, adopted multiscale image fusion following the Laplacian pyramid representation to dehaze the input image. More specifically, the single hazy image and its variants (for example, white-balanced and semi-inverted images) served as input data for the fusion process. Meanwhile, image features, such as saliency, luminance, and chrominance, were exploited to generate guidance maps, which indicated the spatial importance of the input data and thus specified the image regions to be selected for fusion into the final result. Multiscale-fusion-based dehazing algorithms have demonstrated perceptually satisfactory results while maintaining a considerably fast processing speed. Notably, the multiscale fusion technique has broad application in other fields also such as high-quality image generation [9] and low-light image enhancement [10].

**Figure 1:**
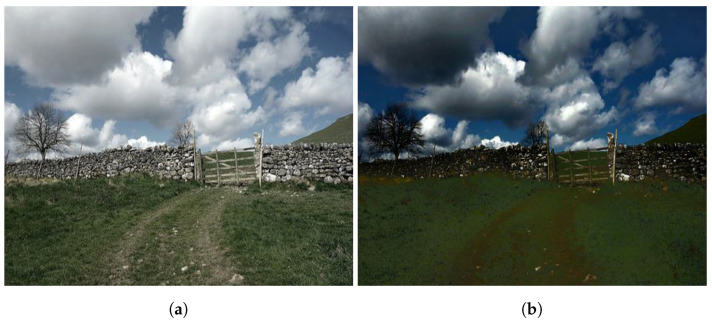
Visibility reduction when invoking image dehazing on a haze-free image. (**a**) Haze-free image and (**b**) its corresponding dehazed result.

Notwithstanding the concerted efforts and momentous achievements to date, dehazing algorithms in the literature are perceived to operate with a “static” approach; that is, they always attempt to remove haze from the input image without even confirming its existence. Accordingly, dehazing a haze-free image results in an apparent loss of visibility, as illustrated in Figure 1, where Figure 1a is a haze-free image, and Figure 1b shows the dehazed version obtained by invoking the well-known algorithm proposed by Zhu et al. [11]. There is a noticeable fading in the fine details of grass and tree twigs, significantly reducing the perceptual visibility. This type of untoward effect is caused by the lack of a specialized evaluator that can inform the dehazing system about the proper time to dehaze the input image. In other words, an automated image dehazing technique is needed that can correctly detect both hazy and haze-free images. In this study, we propose an automated dehazing system (AUDS) with self-calibration on haze conditions to fulfill the aforementioned objective. Given an arbitrary input image, the AUDS produces a dehazed version using a multiscale-fusion scheme. Simultaneously, the AUDS invokes the haziness degree evaluator (HDE) to quantify the image’s haze density, which serves as guidance for ensuring a haze-condition-appropriate dehazing performance. Subsequently, the AUDS employs image blending and tone remapping. The former combines the input image and its dehazed version, and the latter is used to address dynamic range reduction. These two processes are jointly guided by the previously estimated numerical haze density so that the AUDS can attain the automated dehazing objective. In summary, the contributions of this study are as follows:

- An automated scheme is proposed where image dehazing is combined with an HDE to dehaze the input image regardless of the presence of haze.
- Efficient image processing techniques are utilized, such as multiscale fusion, image blending, and tone remapping, to produce satisfactory results.
- A comparative evaluation is performed with well-publicized benchmark methods to demonstrate the efficacy of the proposed AUDS.

The remainder of this paper is organized as follows. Section 2 introduces the hazy image formation, reviews the existing dehazing algorithms in the literature, and describes the motivation for developing the AUDS. Section 3 describes the AUDS in detail by presenting its constituent components. Section 4 provides the comparative experimental assessment results of the AUDS and other state-of-the-art benchmark methods. Finally, Section 5 concludes the study.

## 2. Literature Review

A short introduction to hazy image formation and the results achieved to date in image dehazing is essential for lucidly understanding the concept of AUDS presented later. Accordingly, we first describe the hazy image formation process in the atmosphere and then briefly explore the turning points in the development of image dehazing techniques. Finally, we present the motivation for developing the AUDS to provide an adequate context for Section 3.

### 2.1. Hazy Image Formation

Contemporary researchers typically employ the simplified Koschmieder model [12] to explain hazy image formation in the atmosphere. Under this model, the incoming light waves encounter microscopic particles while traversing the transmission medium to reach the image sensors, inducing atmospheric scattering that increases the captured image luminance. In addition, the reflected light waves from the objects are also subject to attenuation and scattering along the path to the image sensors. However, because modeling all these relevant factors is too complicated, the simplified Koschmieder model does not consider the scattering of the reflected light waves, resulting in the following equation:

(1) I(x)=J(x)t(x)+A[1−t(x)],

where *x* denotes the spatial coordinates of the image pixels, I the hazy image, J the scene radiance, *t* the medium transmittance (also known as the transmission map), and A the global atmospheric light. The boldfaced representation indicates the wavelength-dependent characteristics of the corresponding variables. On the one hand, because digital cameras are typically equipped with red-green-blue (RGB) image sensors, I, J, and A accept values belonging to $R^{H\times W\times 3}$, where *H* and *W* denote the height and width of the image, respectively. On the other hand, *t* is a single-channel variable greater than zero and less than or equal to unity, that is, $t\in R^{H\times W}$ such that 0<t≤1. This variable is depth-dependent and is expressed as t(x)=exp[−βd(x)], where β denotes the atmospheric extinction coefficient and *d* is the scene depth. From this expression, we can observe that the transmittance becomes zero as the scene depth approaches infinity. However, this scenario is non-existent because of the limited imaging technology, which explains the aforementioned claim that 0<t≤1.

The terms J(x)t(x) and A[1−t(x)] are widely referred to as direct attenuation and airlight, respectively. The former denotes the multiplicative attenuation of the reflected light waves in the transmission medium, whereas the latter denotes the additive influence of atmospheric scattering. Accordingly, they are responsible for several hazy image characteristics, such as faint color, reduced contrast, and loss of details. Additionally, the existence of transmittance in these terms indicates its close correlation with the haze density of the images. More specifically, the smaller the transmittance, the denser the haze, and vice versa. Another unknown is the global atmospheric light, A. In the literature, this variable is assumed to be constant as a corollary of the homogeneous-atmosphere postulate. Consequently, A plays a less important role than *t* in the simplified Koschmieder model, which may be why most studies focus on estimating the transmittance or the airlight. The next subsection explores this matter in greater detail.

### 2.2. Related Work

As discussed in Section 1, image dehazing algorithms in the literature fall into two main categories. Whereas those in the first category approach image dehazing from an image enhancement perspective, including traditional histogram equalization and the recently increasing use of image fusion, those in the second adopt an image restoration perspective, modeling the hazy image formation using optical physics and manipulating the model to recover the scene radiance. Hence, this subsection systematically reviews pertinent research and summarizes the major turning points. A systematic review and meta-analysis of this field is available in [13].

Histogram equalization (HE) is a simple and efficient image processing technique with diverse applications, notably image contrast enhancement. Accordingly, researchers have begun leveraging HE to alleviate the undesired atmospheric effects. Among the various HE methods hitherto developed, the block-overlapped HE proposed by Kim et al. [5] is considered efficient and practical because of being verifiable with consumer digital cameras and surveillance cameras. However, researchers have shifted their attention to image fusion because HE-based methods typically fail to consider haze-related degradation. Image dehazing based on image fusion is an elegant solution because of its comparative performance with image-restoration-based methods and fast processing speed. These advantages are accomplished by eliminating the complex estimation of the transmittance and global atmospheric light, as will be discussed later. Recent studies have mainly focused on multiscale fusion (fusing the images on multiple scales). Additionally, although multiscale fusion is achievable by convolving the input image with different-sized kernels, convolving the kernel with the input image and its downscaled versions is more efficient. This idea was presented by Adelson et al. [14], and the word “pyramid” was used to denote the data structure representing the image information. In addition, the name Laplacian pyramid or Gaussian pyramid is derived from the low-pass filtering kernel utilized to downsample the original image.

Ancuti and Ancuti [7] adopted multiscale fusion using the Laplacian pyramid to devise an image dehazing algorithm. They first obtained the white-balanced image and the scaled mean-subtracted image from a single input. After that, they leveraged the image luminance, chrominance, and saliency to compute the corresponding weight maps for image fusion. Finally, the two derived images were fused according to the weight maps using the Laplacian pyramid representation. Although this method can produce satisfactory results, it may fail to handle images with non-uniform lighting conditions (for example, nighttime scenes). Subsequently, Ancuti et al. [8] improved upon the previous work [7] to address the aforementioned issue. By observing that global atmospheric light is an inappropriate assumption for nighttime scenes, they proposed the estimation of the patch-based atmospheric light using two different patch sizes. They interpreted that a small patch size can capture non-uniform lightness, whereas a large patch size is more appropriate for daytime scenes with almost uniform lightness. Consequently, two dehazed versions corresponding to the two patch sizes, coupled with the discrete Laplacian of the input image, were selected as input data for image fusion. The weight maps were derived from the local contrast, saturation, and saliency. The final result was obtained through multiscale fusion according to the Laplacian pyramid representation. The efficacy of multiscale-fusion-based methods is the motivation for developing the dehazing part of the AUDS, and details of this technique will be described in Section 3.2.

An in-depth exploration of image-restoration-based methods is also presented for a comprehensive literature review. In the simplified Koschmieder model, input image (I) is the single observation. Accordingly, the transmittance (*t*) and global atmospheric light (A) are requisites for recovering the scene radiance (J). In the literature, existing algorithms estimate them either separately or jointly (via airlight). The most notable among them is probably the one based on the DCP of He et al. [4]. On the one hand, they estimated the transmittance using two postulates: that the dark channel of the scene radiance approximates zero and that the transmittance is locally uniform. Unfortunately, the second postulate leads to blocking artifacts and requires computationally expensive soft matting [15] for transmittance refinement. On the other hand, He et al. [4] proposed a fairly robust scheme for estimating the global atmospheric light. They first selected the top 0.1 percent brightest pixels in the dark channel and then singled out the highest intensity pixel in the RGB color space as the global atmospheric light. This estimation scheme is relatively robust to the problem of incorrectly selecting bright objects (for example, white cars) instead of the actual lightness. The dehazing approach proposed by He et al. [4] is widely recognized as an efficient algorithm, albeit with two significant problems: high algorithmic complexity and DCP breakdown in sky regions.

Tarel and Hautiere [16] lowered the algorithmic complexity by exploiting the filtering technique known as the median of medians along a line to estimate the airlight. They also postulated that the global atmospheric light is pure white after white-balancing the input image. Consequently, they eliminated the need to estimate the global lightness. As median filtering can be attained in a constant time [17], the algorithm proposed by Tarel and Hautiere [16] exhibits a linear run-time complexity. However, despite the acceleration in the processing rate, this algorithm is prone to halo artifacts because the median filtering unintentionally smoothens the image edges. This problem can be overcome using edge-preserving smoothing filters. The guided image filter [18] and its successors, namely the weighted guided image filter [19] and globally guided image filter [20], are typical examples. These filters are utilized to replace the soft matting in the algorithm proposed by He et al. [4], resulting in significantly reduced algorithmic complexity. This profound benefit is attributed to the local linear model describing the relation between the filtering output and the guidance image. Consequently, the aforementioned filters can transfer the guidance image’s structures to the filtering output while retaining the linear run-time complexity. Similar to Tarel and Hautiere [16], Alajarmeh et al. [21] devised a dehazing framework with linear run-time complexity by virtue of the constant-time atmospheric light and linear-time transmittance estimation. Nevertheless, this method is prone to the color shift problem and incorrect selection of atmospheric light, as demonstrated by the bluish sky in their reported results.

Researchers have also applied machine learning techniques, such as maximum likelihood estimates (MLE) and clustering, to image dehazing. For example, after extensive observations on outdoor images, Zhu et al. [11] discovered the color attenuation prior (CAP), which states that the scene depth correlates with the difference between the image saturation and brightness. Accordingly, the CAP captures this correlation using a linear model and estimates the model parameters through MLE. The transmittance can then be easily calculated based on the scene depth. Meanwhile, the global atmospheric light estimate is obtained utilizing a scheme similar to that of He et al. [4], except that the scene depth replaces the dark channel. However, the CAP-based dehazing algorithm is prone to background noise, color distortion, and post-dehazing false enlargement of white objects. Ngo et al. [22] recently remedied these three problems using low-pass filtering, adaptive weighting, and atmospheric light compensation, respectively. Notwithstanding these improvements, this method delivers an unimpressive performance in dense haze images. Other methods proposed by Tang et al. [23] and Ngo et al. [24] estimate the transmittance from a set of haze-relevant features, using random forest regression and heuristic optimization. Despite the impressive dehazing performance, the two methods are computationally expensive, hindering their broad application. Similarly, Cho et al. [25] estimated the transmittance by maximizing the local contrast while minimizing the number of overshoots and undershoots. Although this method is relatively fast compared with the previous two due to its efficient implementation, its strong dehazing power is subject to color distortion. Another well-recognized strong dehazing algorithm is based on the non-local haze-line prior [26], which states that a few hundred distinct colors can well approximate the true colors of haze-free images. Nevertheless, this method is also prone to color distortion.

Recently, researchers have leveraged deep learning techniques to recover scene radiance in adverse weather conditions. The DehazeNet network, a pioneering attempt by Cai et al. [27], was trained on a synthetic dataset to learn the mapping between the input RGB image and its corresponding transmittance. For this purpose, the DehazeNet architecture comprises three typical layers: feature extraction, multiscale mapping, and nonlinear regression. Subsequent studies have focused on the relaxation of the supervised learning scheme insofar as an unpaired real dataset can now be used to train the network. On the one hand, Li et al. [28] developed a semi-supervised learning framework where the designed network is jointly trained using two branches. The supervised branch follows the typical supervised learning with a paired synthetic dataset, whereas the unsupervised branch only exploits real hazy images to avoid data overfitting. On the other hand, Chaitanya and Mukherjee [29] and Sun et al. [30] exploited a cycle-consistent adversarial network, widely referred to as CycleGAN, to facilitate the use of unpaired datasets. Notably, Li et al. [31] leveraged zero-shot learning, currently in its infancy, to fully relax a paired dataset requirement. They designed three networks that operate on the input image to estimate the scene radiance, transmittance, and global lightness, respectively. They then combined the results to produce an image resembling the original via the simplified Koschmieder model. Thus, this method uses only the input image to train the networks for predicting the scene radiance. However, the zero-shot learning framework substantially prolongs the inference time, impeding real-time processing.

### 2.3. Motivation

The foregoing review summarized the main progress of image dehazing and discussed the major milestones of two approaches: image enhancement and image restoration. Notably, image-restoration-based algorithms face a trade-off between complexity and restoration quality. On the one hand, deep-learning-based dehazing networks typically deliver state-of-the-art performance; however, they require expensive computing platforms for execution. Although Eyeriss-like research [32] on the efficient implementation of deep neural networks has shown a few promising results, they are still inapplicable to deep restoration networks. Real-time processing with energy efficiency is also currently considered unattainable. On the other hand, the dehazing method proposed by Tarel and Hautiere [16] or Zhu et al. [11] is computationally friendly but suffers from other image-quality-related problems, such as color distortion and halo artifacts. Accordingly, in this study, we selected the multiscale fusion technique, whose efficacy in image dehazing has been verified through various studies in the literature.

Moreover, existing algorithms commonly lack a pseudo-cognitive function to dehaze the input image according to its haze density. As illustrated in Figure 1, the haze-free image is significantly degraded when subjected to the dehazing algorithm developed by Zhu et al. [11]. Similar degradation is also observed in other methods mentioned previously. Hence, this observation motivated us to develop the proposed AUDS.

## 3. Proposed System

**Figure 2:**
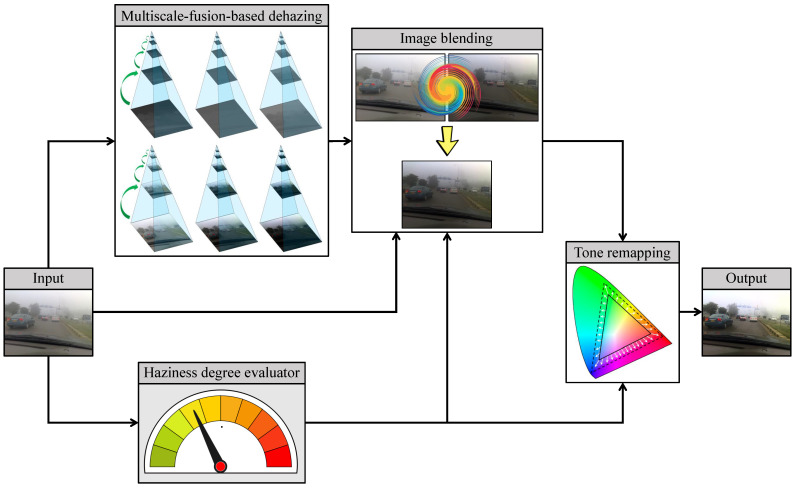
General block diagram of the automated dehazing system (AUDS), founded on multiscale-fusion-based dehazing, haziness degree evaluator, image blending, and tone remapping.

Figure 2 depicts the general block diagram of the proposed AUDS, where the input image undergoes multiscale-fusion-based dehazing and HDE simultaneously. Image blending then combines the dehazed result with the original input according to the haze density estimate. Finally, the blended image undergoes luminance enhancement and color emphasis, conducted within tone remapping. This process is also guided by the haze density estimate, so that the final result exhibits good visibility regardless of the haze condition of the input image. The following subsections describe AUDS’s constituent components in greater detail.

### 3.1. HDE

Researchers have largely ignored the prediction of an image’s haze density, and few studies exist in the literature. Choi et al. [33] developed an indicator known as fog aware density evaluator (FADE), predicting the haze density based on the measurable distances between observed regularities in real hazy and haze-free images. They first collected hazy and haze-free image corpora, each with 500 images. They then extracted twelve haze-relevant features from these image corpora and fitted the features to multivariate Gaussian models to establish corresponding ground-truth references. Thus, the derived mean vectors and covariance matrices represent hazy and haze-free references. The FADE accepts a single image and calculates the deviations from the previous references using Mahalanobis distances, which are subsequently used to derive the haze density estimate. However, the FADE values are not normalized, hindering their application in general circumstances.

Jiang et al. [34] proposed estimating haze density as a polynomial combination of haze-relevant features. Initially, they exploited seven features, resulting in an excessively complex model. They then leveraged sensitivity and error analyses to reduce the model complexity, resulting in a final model with only three features. This model avoids the problems observed in FADE by producing a haze density estimate ranging from zero to unity. It should be noted that the haze density indicators developed by Choi et al. [33] and Jiang et al. [34] are data-driven; that is, the delivered performance depends on the collected image data for offline computation of internal parameters. More specifically, FADE utilizes hazy and haze-free image corpora to establish ground-truth references for haze density estimation, while Jiang et al. [34] relied on the collected data for estimating the model parameters.

Recently, Ngo et al. [35] developed a knowledge-driven approach known as the HDE for haze density estimation. They investigated nine haze-relevant features and selected three, including the product of saturation and brightness, sharpness, and dark channel, using a correlation and computation analysis. These features are computationally efficient and differentiable and are used to formulate an analytically solvable objective function. Optimizing this function yields a closed-form formula for predicting the haze density from a single image. Ngo et al. [35] also conducted a comparative evaluation where the HDE was compared with the aforementioned two evaluators in a hazy/haze-free image classification task. The results demonstrated that the HDE exhibited an accuracy of approximately 96 percent, higher than that obtained using benchmark evaluators and human observers. Additionally, they utilized the HDE as an assessment metric to evaluate the dehazing algorithms. In this case, they compared their experimental results with those previously reported by Galdran [36] and Ancuti et al. [37], validating the superiority of the HDE over FADE. Above all, HDE computation is impressively fast, as evidenced by the run-time comparison with the benchmark evaluators. Hence, we leveraged the HDE in this study to estimate the haze density.

As illustrated by the simplified Koschmieder model, the scene radiance (J) of an image (I) depends on the global atmospheric light (A) and transmittance (*t*). Additionally, because A can be easily obtained from a single input image using the quad-tree decomposition algorithm [38], J is dependent only on *t*. This dependence also applies to the haze-relevant features extracted from J. Accordingly, Ngo et al. [35] formulated the transmittance-dependent objective function [Obj(t)] as follows:

(2) $$Obj\left(t\right)=\frac{SV_{J}\left(t\right)\sigma_{J}\left(t\right)}{J^{dark}\left(t\right)}+\lambda R\left(t\right),$$

where $SV_{J}$ denotes the product of saturation and brightness, $\sigma_{J}$ the sharpness, $J_{dark}$ the dark channel, λ the regularization coefficient, and R(t)=1/t the regularization term. As described above, Ngo et al. [35] selected three haze-relevant features $SV_{J}$, $\sigma_{J}$, and $J_{dark}$, because they are differentiable. Consequently, the optimization problem is analytically solvable, yielding a closed-form formula of the optimal transmittance (denoted as $\hat{t}$), which is fairly lengthy and then is deliberately omitted in this paper. Interested readers are referred to the previous study of Ngo et al. [35] for the full expression. The haze density estimate ($\rho_{I}$) is then derived from $\hat{t}$ using the following equation:

(3) $$\rho_{I}=\frac{1}{\left|\Psi \right|}\sum_{\forall x\in \Psi }\left[1-\hat{t}\left(x\right)\right],$$

where Ψ denotes the entire image domain, |Ψ| the total number of image pixels, and *x* the pixel coordinates within the image (as mentioned in Section 2.1). This estimate ($\rho_{I}$) ranges from zero to unity and is proportional to the haze density.

**Figure 3:**
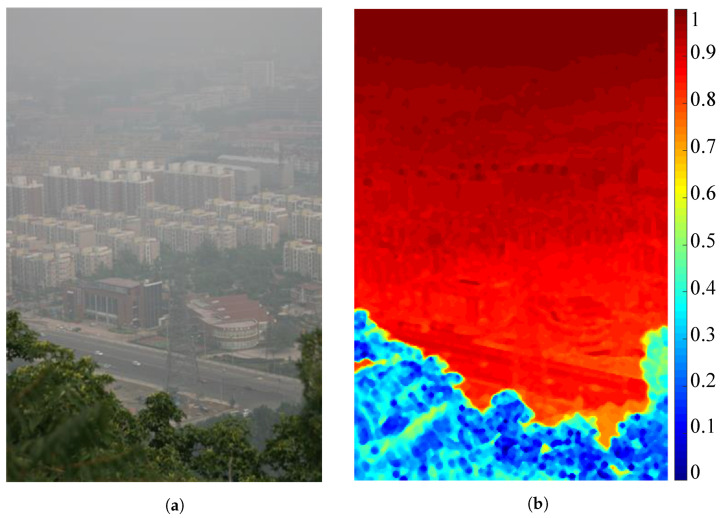
Illustration of haze density estimation. (**a**) Hazy image and (**b**) its corresponding map depicting the local haze density estimates.

Figure 3 demonstrates a hazy image and its corresponding map that represents the local haze density estimates. This map is the term ($1-\hat{t}$) in Equation (3), and its values are normalized for ease of visualization. It can be observed that the map is closely correlated with the actual haze concentration of the hazy image because three distinct hazy regions are easily noticeable. More precisely, Figure 3b demonstrates that the haze concentration increases along with the scene depth, as witnessed by the corresponding increase in the local haze density estimates. For convenience, the proposed AUDS only utilizes the average value $\rho_{I}$ to represent the haze density estimate of the input image. Accordingly, $\rho_{I}$ facilitates the proposed AUDS to guide the image blending and tone remapping processes to obtain a satisfactory result appropriate to the haze condition.

### 3.2. Dehazing Using Under-Exposure and Image Fusion

**Figure 4:**
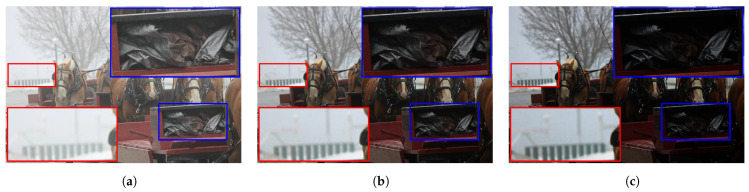
Illustration of under-exposure’s effects on the hazy image. (**a**) Hazy image and its corresponding under-exposed results with (**b**) γ=2 and (**c**) γ=3.

As discussed earlier, multiscale fusion combines several images according to guidance maps to produce an image with desirable characteristics. In single-image dehazing algorithms, a single input is used to obtain multiple images for the fusion process. For that purpose, Galdran [36] proposed the idea of using under-exposed images. However, because under-exposure is related to a physical adjustment of the camera lens to control the light entering the aperture, gamma correction was leveraged to artificially under-expose the input image. This simple technique is expressed by a power-law relationship, where the output varies with the input power. Assuming that the image data are normalized between zero and unity, the power (denoted as γ) defines three operation modes corresponding to γ>1, γ=1, and γ<1, representing under-exposure, non-mapping, and over-exposure, respectively. As illustrated in Figure 4, the under-exposed images obtained with γ=2 and γ=3 exhibit a global reduction in the image intensities. Consequently, objects obscured by haze become noticeable, as depicted in the red-cropped patch. Conversely, the dark details in the blue-cropped patch fade away, which is an undesirable side effect. Hence, selectively fusing these under-exposed images can considerably improve the visibility of hazy images.

Furthermore, Galdran [36] leveraged contrast-limited adaptive histogram equalization (CLAHE) to generate an additional input to the fusion process. This contrast-enhanced input balances the side effect of the gamma correction mentioned above. However, CLAHE may leave blocking artifacts in the dark image. Ngo et al. [39] improved upon the artificial under-exposure by utilizing detail enhancement followed by gamma correction. In this context, the former enhances the object contours obscured by the haze layer, while the latter generates detail-enhanced under-exposed images. The corresponding guidance maps are derived from the dark channel owing to their strong correlation with the haze distribution. Nevertheless, as Ngo et al. [39] concentrated their efforts on real-time hardware design, they merely employed single-scale image fusion. In this study, we adopted a dehazing procedure similar to Ngo et al. [39], except for the use of multiscale fusion. The number of scales (*N*) was set as large as possible, according to Equation (4), to maximize the beneficial effects, wherein min(·) yields a smaller value between image height (*H*) and width (*W*). As a result, the multiscale-fusion-based dehazing process produces a dehazed image (J) directly from the input image (I).

(4) $$N=\lfloor log_{2}\left[min\left(H,W\right)\right]\rfloor .$$

Mathematically, it is assumed that *K* is the number of under-exposed images. The input pyramid is then defined as $\left\{I_{n}^{k}|k,n\in Z,1\leq k\leq K,1\leq n\leq N\right\}$, where images at the first scale (n=1) are obtained using gamma correction, as defined by $\left\{I_{1}^{k}=I^{\gamma_{k}}|\gamma_{k}\in R,\gamma_{k}\geq 1\right\}$. Images at the remaining scales (n>1) are generated using the following equation:

(5) $$I_{n}^{k}=d_{2}\left(I_{n-1}^{k}\right),$$

where $d_{2}\left(\cdot \right)$ denotes the down-sampling operation by a factor of two. Next, the Laplacian pyramid can be constructed by adopting the inversion procedure. At the last scale (n=N), the Laplacian image is defined as $L_{N}^{k}=I_{N}^{k}$. At the remaining scales (n<N), the corresponding Laplacian image is defined by Equation (6), where $u_{2}\left(\cdot \right)$ denotes the up-sampling operation by a factor of two.

(6) $$L_{n}^{k}=I_{n}^{k}-u_{2}\left(I_{n+1}^{k}\right).$$

As introduced previously, we utilized the dark channel prior [4] to calculate the guidance map. For images at the first scale, the corresponding guidance map is defined as below:

(7) $$W_{1}^{k}=1-\min_{y\in \Omega_{MFD}\left(x\right)}\left\{cmin\left[I_{1}^{k}\left(y\right)\right]\right\},$$

where $\min_{y\in \Omega_{MFD}\left(x\right)}\left(\cdot \right)$ denotes the spatially minimum filtering operation over the square patch $\Omega_{MFD}\left(x\right)$, which is centered at the pixel location *x*. Meanwhile, the inner symbol cmin(·) denotes the channel-wise minimum operation. The remaining guidance maps can be easily obtained for images at other scales (n>1) by applying the down-sampling operation recursively, as expressed by $W_{n}^{k}=d_{2}\left(W_{n-1}^{k}\right)$. After that, the guidance maps at individual scales are normalized according to Equation (8) to avoid the out-of-range problem.

(8) $${\tilde{W}}_{n}^{k}=\frac{W_{n}^{k}}{\sum_{k=1}^{K}W_{n}^{k}}.$$

Then, all *K* images are multiplied with *K* corresponding guidance maps at each scale, and the multiplication results are summed up together. Accordingly, this step yields *N* temporary results (denoted as $T_{n}=\sum_{k=1}^{K}{\tilde{W}}_{n}^{k}L_{n}^{k}$) at *N* different scales. Concerning the smallest scale (n=N), the corresponding fusion result is defined as $J_{N}=T_{N}=\sum_{k=1}^{K}{\tilde{W}}_{N}^{k}L_{N}^{k}$. From the (N−1)th scale to the first scale (N−1≥n≥1), the fusion results $J_{n}$ are as follows:

(9) $$J_{n}=u_{2}\left(J_{n+1}\right)\;+\;T_{n}.$$

**Figure 5:**
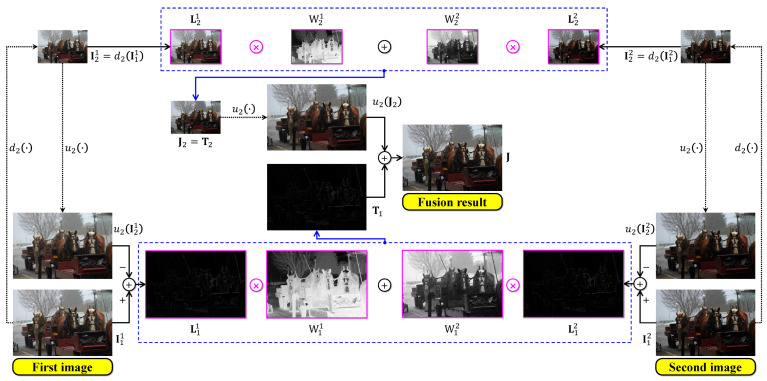
Illustration of multiscale image fusion. The two input images were under-exposed using γ=2 and γ=3. The symbols $u_{2}\left(\cdot \right)$ and $d_{2}\left(\cdot \right)$ denote the up- and down-sampling operations by a factor of two.

Generally, the desirable fusion result is the one at the first scale, that is, $J_{1}$. So, the dehazed image produced by the multiscale-fusion-based dehazing method is $J=J_{1}$. Figure 5 below demonstrates a simple multiscale fusion process using two under-exposed images illustrated in Figure 4b,c. These two images are denoted as $I_{1}^{1}$ and $I_{1}^{2}$, respectively. It can be observed that $I_{1}^{1}$ possesses a clearer foreground than $I_{2}^{1}$, whereas its background is hazier than that of $I_{2}^{1}$. As a result, the corresponding guidance maps demonstrate that the fusion process combines the foreground of $I_{1}^{1}$ with the background of $I_{2}^{1}$, yielding a satisfactorily fused image with improved visibility.

The following image blending process operates on the input image (I), haze density estimate ($\rho_{I}$), and dehazed image (J) to produce a blended image unaffected by weather-related degradation. In this context, the $\rho_{I}$ value is used to generate a haze-condition-appropriate ratio for mixing I and J; hence, image blending is self-calibrated.

### 3.3. Image Blending

Image blending is a simple technique wherein the color values of two images are combined according to a pre-determined percentage. The result then exhibits the desirable characteristics of the two source images. In other words, image blending can be considered a special case of image fusion, where the fusion is conducted on a single scale using constant weight maps. This simple technique has also been utilized for frame interpolation in early television technology. Accordingly, we leveraged the image blending technique to combine the original input (I) and its dehazed version (J) to obtain a hazy-weather-unaffected result. The corresponding amalgamation ratio of I to J is determined using the hazy density estimate ($\rho_{I}$) obtained via the HDE’s execution on I. As an intuitive description, we relied on $\rho_{I}$ to classify the input image as “haze-free”, “mildly hazy”, “moderately hazy”, or “densely hazy”. The image blending step results in one of the following outputs:

- An input image if the haze condition is “haze-free”.
- A dehazed image if the haze condition is “densely hazy”.
- A linear combination of the input image and its dehazed version if the haze condition is either “mildly hazy” or “moderately hazy”.

As $\rho_{I}$ lies between zero and unity, two user-defined thresholds, $\rho_{1}$ and $\rho_{2}$, can be used to divide the value range into three regions: haze-free, hazy, and densely hazy, corresponding to $\rho_{I}<\rho_{1}$, $\rho_{1}\leq \rho_{I}\leq \rho_{2}$, and $\rho_{I}>\rho_{2}$, respectively. Hazy images are then further classified into mildly and moderately hazy, consistent with the haze conditions defined above. On the one hand, we utilized the threshold value determined by Ngo et al. [35] as $\rho_{1}$ because it has been used to classify hazy/haze-free images. On the other hand, the value of $\rho_{2}$ was determined as the mean HDE value of the hazy image corpora, as summarized in Table 1. IVC [40], FRIDA2 [41], D-HAZY [37], O-HAZE [42], I-HAZE [43], and Dense-Haze [44] are well-publicized datasets that are widely used to evaluate image dehazing algorithms. FINEDUST [24] and 500IMG [22], in contrast, are self-collected datasets from our previous work. Therefore, the values of $\rho_{1}=0.8811$ and $\rho_{2}=0.9344$ were used in this study to produce the results presented later. Additionally, the aforementioned image blending output can be rewritten as follows:

- The blended result is the input image if $\rho_{I}<\rho_{1}$.
- The blended result is the dehazed image if $\rho_{I}>\rho_{2}$.
- The blended result is a linear combination of these two images if $\rho_{1}\leq \rho_{I}\leq \rho_{2}$.

For the two extremes ($\rho_{I}<\rho_{1}$ and $\rho_{I}>\rho_{2}$), the proposed AUDS classifies the input image as haze-free or densely hazy. It then maximizes or minimizes the contribution of the input image to the blended result accordingly. In other words, the AUDS sets the percentages for the input image and its dehazed version in the image blending step to (100%,0%) or (0%,100%), respectively. In the remaining case ($\rho_{1}\leq \rho_{I}\leq \rho_{2}$), the AUDS treats the input image as mildly or moderately hazy. It then determines the blending percentages so that both the input image and its dehazed version contribute to the blended result. Therefore, it is essential to map the values of $\left[\rho_{1},\rho_{2}\right]$ to [0,1] for facilitating this determination. The mapping is conducted by applying Equation (10), where ${\hat{\rho }}_{I}$ denotes the mapped values of $\rho_{I}$, and α is a user-defined positive parameter controlling the shape of the mapping curve. For haze-free and densely hazy images, the corresponding ${\hat{\rho }}_{I}$ values are mapped to zero and unity, respectively. For hazy images whose $\rho_{I}$ values lie between the two thresholds, the mapping is generalized using α while ensuring that the corresponding ${\hat{\rho }}_{I}$ values range from zero to unity. As will be described shortly, α controls the degree to which the image blending transfers the input image and its dehazed version to the blended result.

(10) $${\hat{\rho }}_{I}=\{\begin{array}{cc} 0 & \rho_{I}<\rho_{1}\\ {\left(\frac{\rho_{I}-\rho_{1}}{\rho_{2}-\rho_{1}}\right)}^{\alpha } & \rho_{1}\leq \rho_{I}\leq \rho_{2}.\\ 1 & \rho_{I}>\rho_{2} \end{array}$$

**Figure 6:**
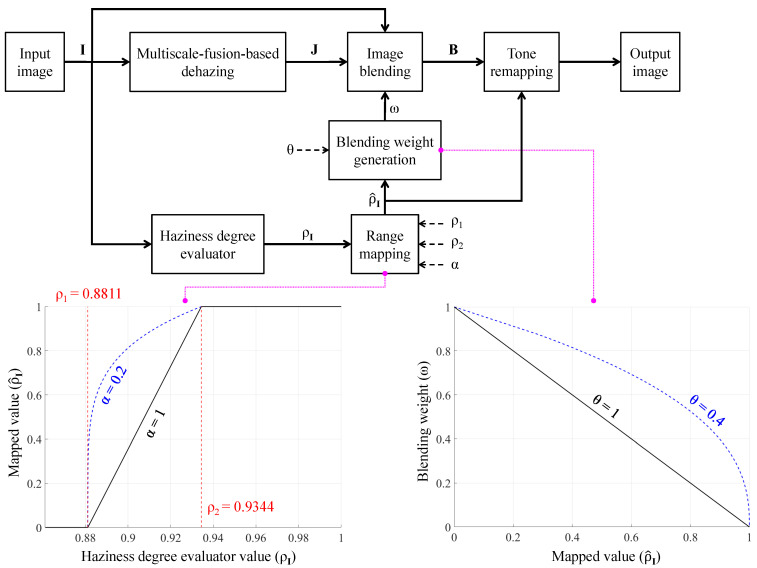
Detailed block diagram of the AUDS demonstrating the exploitation of haziness degree evaluator to guide image blending and tone remapping.

The blending weight (or percentage) is then generated from ${\hat{\rho }}_{I}$ as follows:

(11) $$\omega ={\left(1-{\hat{\rho }}_{I}\right)}^{\theta },$$

where ω denotes the blending weight associated with the input image (I), and θ is a user-defined positive parameter controlling the contribution ratio of the involved images. The image blending step is then conducted using Equation (12), where B denotes the blended result. Figure 6 provides a more intelligible description of the general block diagram in Figure 2 by incorporating the information presented so far, including two graphs for Equations (10) and (11), respectively. As the blending step combines the input image and its corresponding dehazed version, the contribution ratio is crucial for producing satisfactory results. However, the highly subjective perception of image quality induced us to use two user-defined parameters α and θ. The former implicitly controls the contribution ratio via ${\hat{\rho }}_{I}$, whereas the latter’s effect is explicit, as shown in Equation (11). Accordingly, users can fine-tune the AUDS to obtain preferable results. For example, the AUDS may exhibit a weak dehazing power on bright and mildly hazy images because bright details are affected by both haze and probable over-illumination. Therefore, a viable solution is to increase the contribution of the dehazed image under these circumstances. As illustrated in Figure 6, the mapping curve corresponding to α=0.2 was utilized in this study to yield high ${\hat{\rho }}_{I}$ values for mildly hazy images. Accordingly, the blending weight generation, expressed by Equation (11), results in low ω values, signifying that more information from the dehazed image is transferred into the blended result. It should also be noted that the three terms—percentage, weight, and ratio—and their corresponding representations have been used interchangeably in this study. For example, the blending percentages (70%,30%) are analogous to the blending weights (0.7,0.3) and the contribution ratio 7:3.

(12) B=ωI+(1−ω)J.

As described in Section 4.4, we assumed an empirical configuration of (α,θ)=(0.2,0.4) after extensive experiments on both real and synthetic image datasets. The corresponding curves representing the range mapping and blending weight generation are illustrated by the dashed blue lines in Figure 6. If the numerical haze density of the input image is less than $\rho_{1}$ (that is, the input image is haze-free), the mapped value ${\hat{\rho }}_{I}$ is zero. This value results in the weight ω=1; hence, B=I signifies that the blended result is the input image. Conversely, if the numerical haze density of the input image is greater than $\rho_{2}$ (that is, the input image is densely hazy), the mapped value is one. This value results in the weight ω=0, signifying that the blended result is the dehazed image, that is, B=J. In the final case, when the numerical haze density of the input image falls between $\rho_{1}$ and $\rho_{2}$ (that is, the input image is mildly or moderately hazy), the mapped value ranges between zero and unity. The higher mapped value results in a smaller weight, signifying that the contribution of the input image to the blended result is less than that of its dehazed version. Therefore, the proposed AUDS delivers satisfactory performance under all haze conditions. We utilized the aforementioned configuration of (α,θ) in this study to ensure that the contribution ratio of the input image and its dehazed version under mildly and moderately hazy images was approximately 7:3 and 5:5, respectively.

Furthermore, Figure 6 illustrates that the mapped value (${\hat{\rho }}_{I}$) is also input to the tone remapping block. The next subsection describes how this value is utilized to guide the tone remapping process in more detail.

### 3.4. Tone Remapping

Image dehazing is fundamentally the subtraction of the haze layer from the input image. Therefore, the dehazed image is typically prone to dynamic range reduction, probably caused by overflows and underflows resulting from arithmetic operations in the restoration process. Hence, tone remapping is considered an efficient post-processing step for extending the reduced dynamic range. We leveraged the adaptive tone remapping (ATR) proposed by Cho et al. [45] to perform both luminance enhancement and color emphasis. These two operations are mathematically defined in Equations (13) and (14), where $L_{e}$ denotes the enhanced luminance, *L* the luminance derived from the blended image B, $G_{L}$ the luminance gain defined as a nonlinear function based on the cumulative distribution of *L*, and $W_{L}$ the adaptive luminance weight defined as a simple linear function of *L*. A similar description is applicable to color emphasis in Equation (14), except that the constant 0.5 denotes an offset to convert the zero-centered chrominance back to the normalized range.

(13) $$\begin{array}{ccc} L_{e}\left(x\right) & = & L\left(x\right)+G_{L}\left(x\right)W_{L}\left(x\right), \end{array}$$

(14) $$\begin{array}{ccc} C_{e}\left(x\right) & = & C\left(x\right)+G_{C}\left(x\right)W_{C}\left(x\right)+0.5. \end{array}$$

We utilized the mapped value ${\hat{\rho }}_{I}$ calculated previously to modify the ATR so that this post-processing step is also guided (or self-calibrated) by the haze condition of the input image. As Cho et al. [45] conducted color emphasis in proportion to the luminance enhancement by defining $G_{C}=C\cdot L_{e}/L$, modifying the adaptive luminance weight $W_{L}$ suffices for the stated purpose. Specifically, ${\hat{\rho }}_{I}$ is multiplied with the adaptive luminance weight such that $W_{L}$ in Equation (13) is replaced by ${\hat{\rho }}_{I}W_{L}$. As a result, if the input image is haze-free (${\hat{\rho }}_{I}=0$), the modified adaptive weight becomes zero, allowing the ATR to bypass the blended image, which is the original haze-free image. In contrast, if the input image is densely hazy (${\hat{\rho }}_{I}=1$), the ATR fully performs luminance enhancement and color emphasis, as described by Equations (13) and (14), respectively. If the input image is mildly or moderately hazy ($0<{\hat{\rho }}_{I}<1$), the added term ${\hat{\rho }}_{I}$ modifies the adaptive weight $W_{L}$ according to the haze density. Therefore, the ATR appropriately enhances the luminance and emphasizes the color insomuch that the final result exhibits satisfactory enhancement quality. Mathematically, the image’s haze density is proportional to the value estimated by the HDE ($\rho_{I}$) and its mapped value ${\hat{\rho }}_{I}$. As a result, the denser the haze, the larger the ${\hat{\rho }}_{I}$ value, which increases the contribution of the dehazed image to the blended result. This increase darkens the blended image and may cause untoward distortion. Hence, the modified adaptive weight ${\hat{\rho }}_{I}W_{L}$ can compensate for this problem by using the mapped value ${\hat{\rho }}_{I}$.

## 4. Experimental Results

This section presents the results of the experimental assessment conducted on the proposed AUDS and other state-of-the-art benchmark methods, including those proposed by Tarel and Hautiere [16], He et al. [4], Ngo et al. [39], Zhu et al. [11], Berman et al. [26], Cho et al. [25], Cai et al. [27], and Ren et al. [46], in terms of qualitative and quantitative performance. In the upcoming sections, we will refer to these benchmark methods by their abbreviation, which can found in Table 2.

### 4.1. Parameter Configuration

The proposed AUDS comprises four main components as illustrated in Figure 2: multiscale-fusion-based dehazing, HDE, image blending, and tone remapping. Each constituent component, in turn, can be configured using a particular set of parameters. Therefore, this subsection summarizes the AUDS parameters and provides their corresponding empirical values to help researchers to reproduce the presented experimental results.

As mentioned in Section 3.2, we leveraged the image-fusion-based dehazing method proposed by Ngo et al. [39], except the multiscale approach to image fusion. This image dehazing method comprises the following operations: sharpness enhancement, artificial under-exposure via gamma correction, guidance weight generation, and image fusion. First, the sharpness enhancement is conducted on the input image according to the local variance, which is exploited to determine the degradation degree of local patches. In this context, a pair of variance thresholds $\left(\nu_{1},\nu_{2}\right)$ defines the three degradation degrees: heavy, moderate, and slight. A corresponding pair of scaling factors $\left(\kappa_{1},\kappa_{2}\right)$ then defines the extent to which sharpness enhancement will be conducted. In this study, we utilized the empirical values suggested by Ngo et al. [39] to configure the four parameters $\left\{\nu_{1},\nu_{2},\kappa_{1},\kappa_{2}\right\}$. Next, the artificial under-exposure is attained via gamma correction, expressed by a power-law relation $b=a^{\gamma }$, where *a* and *b* denote image data normalized to the range [0,1], and the positive values γ≥1 denote under-exposure. At this stage, the number of artificially under-exposed images *K* must be defined first. The corresponding values $\gamma_{k}$, where k∈Z∩[1,K], are then determined to perform the gamma correction. The quality of the dehazed image is proportional to the number of artificially under-exposed images *K*. However, *K* is constrained by the limited representation of discrete data in digital systems. In other words, gamma values $\gamma_{k}$ are upper-bounded because very small image intensities resulting from large gamma values may be represented by the same quantization level. Therefore, in this study, we empirically determined K=8 and the corresponding gamma values $\gamma_{k}$ where k∈Z∩[1,8]. In the next step of guidance weight generation, the single user-defined parameter is the patch size $\Omega_{MFD}$ to calculate the dark channel.

The HDE for estimating the image’s haze density is equipped with three user-defined parameters: regularization coefficient λ, emphasis strength $\gamma_{HDE}$, and patch size $\Omega_{HDE}$. The regularization coefficient is introduced into the HDE to ensure that the estimated haze density lies between zero and unity. Meanwhile, the power-law expression $b=a^{\gamma }$ mentioned above is leveraged for its proven performance boost to the HDE, resulting in a remarkable accuracy of 96 percent in the hazy/haze-free image classification tasks [35]. It is also necessary to define the patch size because the HDE computation includes filtering operations resulting from the involvement of the dark channel and sharpness.

The remaining two components (that is, image blending and tone remapping) are guided by the haze density estimate resulting from the HDE’s execution. This procedure involves four user-defined parameters, including a pair of thresholds $\left(\rho_{1},\rho_{2}\right)$ and two power values (α,θ). These parameters are used for range mapping and blending weight generation, as described in Section 3.3. Table 3 provides a summary of all the user-defined parameters and their corresponding empirical values. This parameter configuration was used to generate all the experimental results discussed later.

Concerning other benchmark algorithms, their authors also share the source codes together with corresponding parameter configurations for reproducibility. Therefore, we utilized these software implementations and retained the provided parameter configurations in the following performance assessment.

### 4.2. Qualitative Comparison of Hazy Images

This subsection discusses the comparative dehazing performance of the proposed AUDS and eight benchmark methods on mildly, moderately, and densely hazy images from the eight datasets listed in Table 1. The AUDS, equipped with the HDE, can quantify the image’s haze density and then guide the image blending and tone remapping processes to generate a haze-condition-appropriate result. This simple but efficient addition gives the AUDS a definite advantage over benchmark methods. Specifically, FFD, DCP, NLD, and MBD generally exhibit strong dehazing power, which is beneficial for densely hazy images but not for mildly and moderately hazy images. Notably, in the results of FFD, halo artifacts manifest themselves around fine edges, significantly impairing the image quality. The machine-learning-based CAP also overly dehazes mildly and moderately hazy images, causing color distortion and a probable loss of dark details. Meanwhile, the deep-learning-based DehazeNet and MSCNN can alleviate the previously observed problems to a certain extent, attributable to the powerful representation capability of deep neural networks. These two methods can extract various image features and combine them nonlinearly to estimate the medium transmittance; hence, they can handle images with various haze conditions. Nevertheless, they are prone to the domain shift problem owing to the lack of real training datasets. This observation is validated by the qualitative evaluation results presented below.

**Figure 7:**
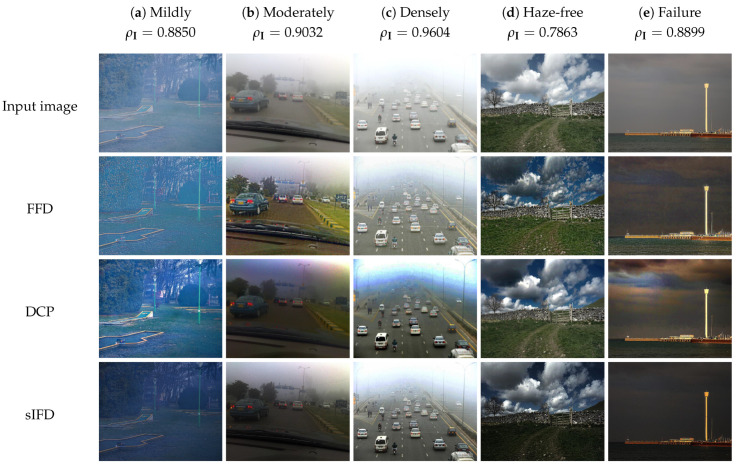

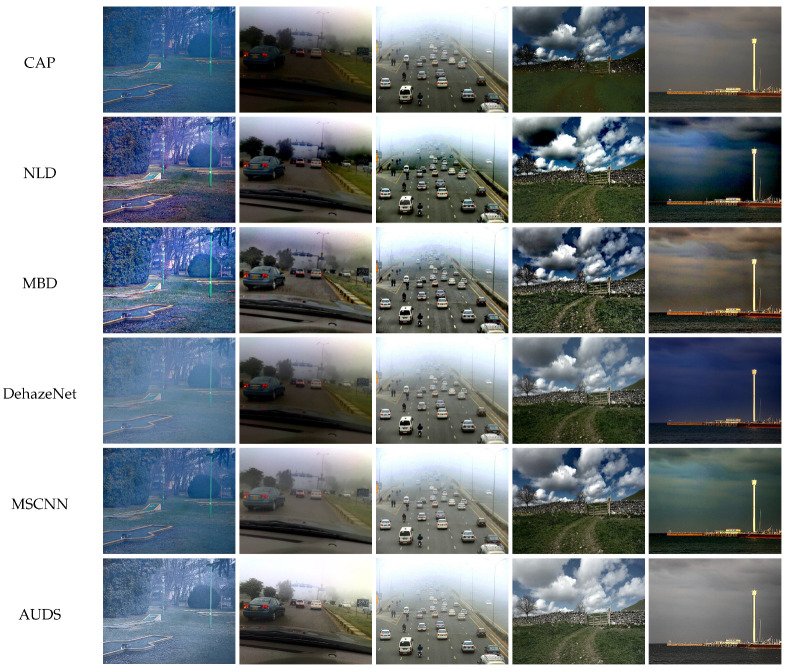
Qualitative comparison of the proposed AUDS with state-of-the-art benchmark methods on different images. Results of FFD, DCP, CAP, DehazeNet, and MSCNN in the (**b**) column are adopted from Ngo et al. [13].

Figure 7 illustrates the qualitative comparison results under different haze conditions. The numerical haze density $\rho_{I}$ can be considered in conjunction with the threshold values $\left\{\rho_{1},\rho_{2}\right\}=\left\{0.8811,0.9344\right\}$ to verify the haze condition of the corresponding input image. For this comparison, we consider the first three columns of the figure that depict real scenes degraded by mild, moderate, and dense haze. It should be noted that results of FFD, DCP, CAP, DehazeNet, and MSCNN in the second column are adopted from Ngo et al. [13].

The results of FFD, DCP, CAP, NLD, and MBD exhibit different types of distortion, ranging from the less noticeable loss of details to the apparent color distortion or halo artifacts. The results of DehazeNet and MSCNN, in contrast, are more favorable to human perception because of a significant reduction in dehazing-induced side effects. However, despite exploiting computation-intensive deep models, the untoward distortion persists, albeit not as severely as those in previous methods. For sIFD and the proposed AUDS, it can be observed that they deliver acceptable performance in all three cases, although their dehazing power is not as strong as that of NLD and MBD. Additionally, the results of AUDS exhibit higher visibility than those of sIFD, attributed to the self-calibrated image blending and tone remapping. The HDE is highly accurate, resulting in an appropriate weight for guiding those two processes to produce a desirable result. As illustrated in the last row of Figure 7, the dehazing results are visually satisfactory without any unpleasant artifacts.

### 4.3. Qualitative Comparison of Haze-Free Images

This subsection completes the aforementioned comparison by considering the case of haze-free input images. As benchmark methods lack the ability to detect the existence of haze, they dehaze even the haze-free images, resulting in an apparent degradation in image visibility. For example, the fourth column of Figure 7 demonstrates a qualitative comparison of a real haze-free scene. Except for the proposed AUDS, all eight benchmark methods exhibit dehazing-induced degradation, such as loss of dark details or color distortion. Specifically, the results of FFD, DCP, sIFD, CAP, NLD, and MBD exhibit a significant reduction in image intensity, causing a loss of dark details. In contrast, comparatively milder problems are observed in the results of DehazeNet and MSCNN, which is attributed to a large number of informative features learned by the deep neural network. However, this type of degradation poses certain practical difficulties in high-level vision applications such as object recognition, localization, and smart surveillance.

Unlike benchmark methods, the proposed AUDS is haze-aware; that is, it can perceive the existence of haze in input images and perform image dehazing appropriately. This desirable course of action results from using the HDE to jointly guide the image blending and tone remapping processes. In particular, image blending combines the input image and its dehazed version in a specific ratio determined by the haze density estimate. For example, the proposed AUDS correctly classifies the input image shown in the fourth column of Figure 7 as a haze-free image because the haze density estimate $\rho_{I}=0.7863$ is less than the threshold $\rho_{1}=0.8811$. Accordingly, the blending weights are set as (1,0) to transfer the entire input image into the blended result. Additionally, as the mapped value ${\hat{\rho }}_{I}$ is zero, the tone remapping step bypasses the blended result, keeping the input image unchanged throughout the proposed AUDS.

Nevertheless, Ngo et al. [35] discovered that the HDE incorrectly classifies images with large and homogeneous backgrounds (for example, sky, sea, and lake) as hazy images. Owing to this drawback of the HDE, image blending and tone remapping may transfer most of the dehazed image into the final result, possibly causing untoward degradation, such as color distortion and a loss of dark details. For example, in the last column of Figure 7, although the input image is haze-free, it contains a large dim sky. Consequently, the HDE incorrectly quantifies its haze density as $\rho_{I}=0.8899$, which is larger than the threshold $\rho_{1}=0.8811$. Hence, the haze-free image is considered a mildly hazy image, and the proposed AUDS attempts to restore the image visibility instead of leaving it unchanged. However, because the HDE classifies the input image as a mildly hazy image, the result does not exhibit any noticeable degradation. Notably, the results of the proposed AUDS demonstrate clearer visibility compared with the input image. In contrast, other benchmark methods display undesirable degradation to different extents, ranging from the slight luminance reduction in the result of CAP to severe color distortion in those of FFD, DCP, sIFD, NLD, MBD, DehazeNet, and MSCNN. Hence, the qualitative comparison results in Figure 7 demonstrate the superiority of AUDS over the eight benchmark methods with five cases: (a) mildly, (b) moderately, (c) densely, (d) haze-free, and (e) failure.

### 4.4. Quantitative Comparison

Subjective ratings obtained from human observers provide the most accurate evaluation of image processing algorithms. However, despite the high reliability, obtaining subject-rated scores is a laborious and unrepeatable task. Therefore, image quality assessment (IQA) metrics have been developed to address this problem. Although IQA metrics do not necessarily correspond to human visual standards, they are adequately reliable. Additionally, an objective assessment using IQA metrics, coupled with the aforementioned qualitative assessment, is widely considered a thorough evaluation.

In this study, we utilized the tone-mapped image quality index (TMQI) and the feature similarity index extended to color images (FSIMc), proposed by Yeganeh and Wang [47] and Zhang et al. [48], respectively, to quantitatively assess the dehazing performance of all nine methods. The TMQI assesses the multiscale structural fidelity and statistical naturalness, and TMQI values are bounded between zero and unity. Higher TMQI values signify that the dynamic range of the restored image shows greater resemblance with that of the ground-truth image. The FSIMc can be considered an upgrade of the structural similarity proposed by Wang et al. [49] because it incorporates chrominance into its computation. This IQA metric is also bounded between zero and unity, with a preference for high values in image restoration tasks.

Table 4 tabulates the quantitative results on real (O-HAZE, I-HAZE, Dense-Haze, and 500IMG) and synthetic (FRIDA2 and D-HAZY) datasets. Except the 500IMG, these datasets contain hazy images and their corresponding haze-free references. Firstly, concerning hazy images, the performance delivered by the proposed AUDS is comparable with that of the best-performing method in each dataset. More specifically, on FRIDA2, D-HAZY, O-HAZE, I-HAZE, and Dense-Haze datasets, the proposed AUDS demonstrates FSIMc scores lower than those of the corresponding best method by 0.14%, 3.52%, 0.87%, 0.42%, and 14.24%, respectively. Meanwhile, the differences in TMQI scores are 6.00%, 10.67%, 0.09%, 1.51%, and 9.43%. As a result, when considering all hazy images from these five datasets, the proposed AUDS is ranked first and second under FSIMc and TMQI metrics, respectively. This observation is explicable because the proposed AUDS is designed to handle various haze conditions. Therefore, it may not deliver excellent performance on a particular haze condition, but the result, in general, is always satisfactory. Additionally, the quantitative evaluation results on hazy images imply a trade-off between dehazing power and dynamic range, represented by FSIMc and TMQI metrics, respectively. Algorithms that exhibit strong dehazing power are subject to undershoots, which cause the pixel value to be black-limited and reduce the dynamic range.

Furthermore, although most studies in the literature have reported using hazy images to conduct a comparative evaluation, the input images for a particular dehazing system may not necessarily be hazy in the real world. Therefore, we also assess nine algorithms using haze-free images. As expected, eight benchmark algorithms deliver unsatisfactory performance because they apply the same dehazing procedure on haze-free images. Consequently, the dehazing results are subject to visually untoward artifacts, such as color distortion and loss of fine details, as illustrated in Figure 1. Conversely, the proposed AUDS is equipped with the HDE to perceive whether the haze condition of the input image. After that, it modifies the image blending and tone remapping correspondingly to bypass the haze-free image. This appropriate course of action yields excellent performance, as demonstrated in Table 4. More precisely, the proposed AUDS is ranked first on all datasets in terms of FSIMc metric, while it only retains its superiority on real datasets in terms of TMQI metric. On synthetic datasets (FRIDA2 and D-HAZY), a few haze-free images exhibit a broad sky—which is pure white—in the background, deceiving the HDE into misclassifying them as densely hazy images. Accordingly, the proposed AUDS fuses the dehazed result into the final image, reducing the performance in this case. Additionally, the fact that the sIFD outperforms the proposed AUDS on these two synthetic datasets is due to the difference between single-scale and multiscale image fusion. On hazy images, multiscale processing is beneficial for bringing out details obscured by the haze layer. However, this technique may cause untoward artifacts on haze-free images, which are less noticeable when processing on the original scale.

Overall, the proposed AUDS is well-performed on various haze conditions, whereas other benchmark algorithms exhibit poor performance on haze-free images. This superiority is mainly attributed to the HDE guidance in image blending and tone remapping processes. With a high classification accuracy (96 percent), the HDE is generally very accurate in identifying haze-free input images. The derived weights then appropriately guide the image blending and tone remapping processes to transfer the input image into the final result. In contrast, benchmark methods cannot perform image dehazing in a haze-density-adaptive manner, causing undesirable degradation, such as color distortion and a loss of dark details. Consequently, the corresponding results on haze-free images are relatively poor, lowering the overall performance and widening the performance gap with the proposed AUDS. This observation is evident in the last row of Table 4, where the best results are boldfaced in red, green, and blue in descending order.

**Figure 8:**
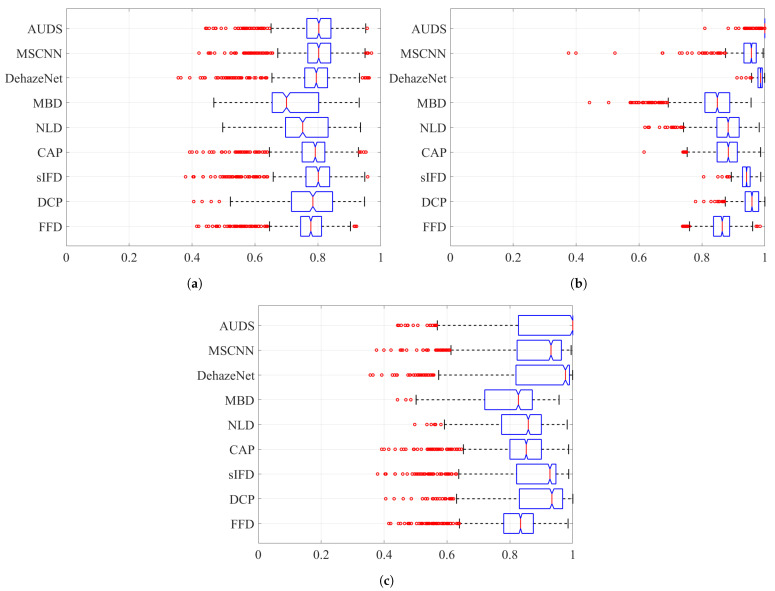
Boxplots of FSIMc scores on different datasets when considering: (**a**) hazy images only, (**b**) haze-free images only, and (**c**) both hazy and haze-free images.

**Figure 9:**
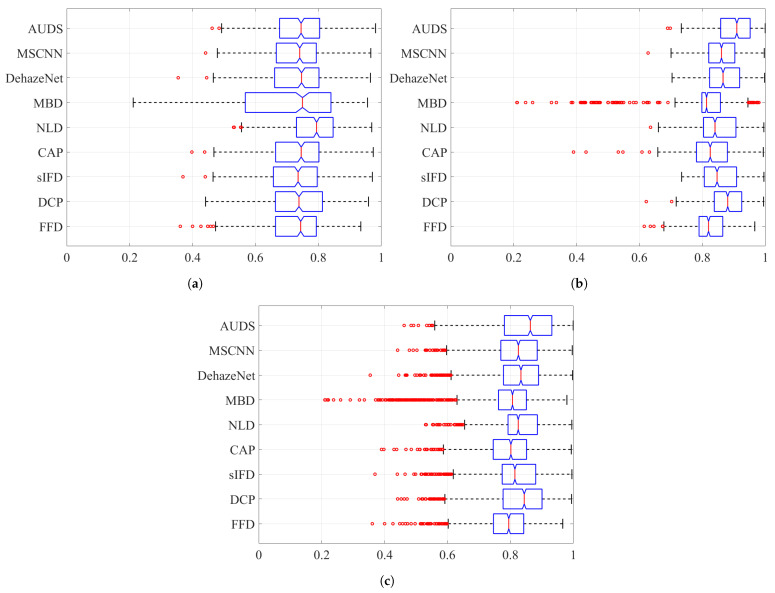
Boxplots of TMQI scores on different datasets when considering: (**a**) hazy images only, (**b**) haze-free images only, and (**c**) both hazy and haze-free images.

Next, Figure 8 and Figure 9 depict the boxplots of the FSIMc and TMQI scores to provide more insights into the quantitative evaluation. It should be noted that most of the possible outliers (depicted as red round dots) are from the Dense-Haze dataset, whose constituent images are affected by dense haze, hence the low FSIMc and TMQI scores. In Figure 8a, it can be observed that the proposed AUDS exhibits the highest median, followed by MSCNN. Additionally, these medians do not overlap with those for other methods, signifying that the medians are statistically different with 95% confidence. Concerning the spread and possible outliers, Figure 8a demonstrates no significant difference between FFD, sIFD, CAP, DehazeNet, MSCNN, and the proposed AUDS. Their FSIMc scores roughly range from 0.65 to 0.95. Meanwhile, DCP, NLD, and MBD also exhibit some resemblance in their overall spread of values, which is broader than that of the remaining methods. This observation signifies that the strong dehazing power of DCP, NLD, and MBD renders them prone to post-dehazing artifacts, which lower their FSIMc scores. The conclusion drawn from Figure 8a is similar to the previous one based on the average FSIMc scores in Table 4.

Concerning haze-free images, the boxplots in Figure 8b demonstrate the superiority of the proposed AUDS conspicuously. As the HDE discriminates the haze condition accurately, the proposed AUDS can handle haze-free images effectively. Accordingly, Figure 8b shows that the median for AUDS is very close to unity with the extremely narrow spread and notch, offering statistically significant evidence of the difference between the medians. Thus, the excellent performance on haze-free images improves the overall performance on both hazy and haze-free images, as illustrated in Figure 8c.

Considering the TMQI scores, Figure 9a demonstrates that NLD outperforms other methods on hazy images per se. This observation is statistically backed by a clear distinction between its median and that of other methods. In addition, except for MBD with too broad a spread of TMQI scores, FFD, DCP, sIFD, CAP, NLD, DehazeNet, MSCNN, and AUDS exhibit similar distributions. Figure 9b demonstrates the pre-eminence of AUDS over eight benchmark methods on haze-free images, similar to the interpretation on the boxplots in Figure 8. Hence, the best overall performance of the proposed AUDS under the TMQI metric is also backed by statistical evidence.

### 4.5. Run-Time Comparison

According to the description in Section 3, the proposed AUDS is mainly composed of simple operations that have linear-time complexity except for the spatial image filter. Conventionally, filtering an H×W image by a sv×sv kernel takes O(H·W·sv·sv) time. Therefore, several studies have been witnessed on the fast implementation of commonly used spatial image filters, such as minimum/maximum filter [50], box filter [51], and median filter [17]. As a result, the spatially filtering operations in the proposed AUDS can be implemented in O(H·W) time; hence, the linear-time complexity of the proposed AUDS.

Table 5 demonstrates a run-time comparison between the nine algorithms mentioned above. The simulation environment is MATLAB R2019a, running on a computer with an Intel Core i9-9900K (3.6 GHz) CPU, 64 GB RAM, and NVIDIA TITAN RTX GPU. Input images with various resolutions were used as test images. From the comparison results, nine algorithms can be approximately classified into three groups: slow, passable, and fast. The slow group includes DCP, NLD, and DehazeNet. In contrast, CAP, sIFD, and FFD exhibit the fastest processing speed and belong to the fast group. The proposed AUDS improves sIFD with three processes: haze density estimation, image blending, and tone remapping. Additionally, Ngo et al. [39] only conducted image fusion at the original scale to facilitate the hardware implementation, whereas the proposed AUDS performed image fusion from the smallest possible scale to the original scale. Accordingly, the proposed AUDS requires more time than the base method to process images, and Table 5 shows that the processing time has been nearly tripled. For that reason, the proposed AUDS falls into the passable group with MBD and MSCNN.

Moreover, the fact that the proposed AUDS is slower than the deep-learning-based method of Ren et al. [46] merits an explanation. The core of this method is the multi-scale convolutional neural network for estimating the transmittance, and Ren et al. [46] implemented this network using the open-source MatConvNet toolbox [52]. As most of MatConvNet’s building blocks are written in C++ and well-optimized, this method is relatively fast compared with the typical implementation of deep neural networks on MATLAB. The proposed AUDS, by contrast, was implemented using MATLAB’s building functions; hence, the slow processing speed compared with C++ implementation is an inherent problem. Nevertheless, the run-time comparison demonstrates that achieving real-time processing with computational efficiency is challenging for all nine algorithms. Addressing this issue is effort-intensive and thus is left for future studies.

## 5. Conclusions

This paper presents a novel approach for dehazing a single image, regardless of haze conditions. The proposed AUDS is equipped with a pseudo-cognitive function realized by the HDE to perceive the image haze density. Accordingly, the input image and its dehazed version obtained via multiscale-fusion-based dehazing are combined using image blending and then post-processed with tone remapping. These two processes are self-calibrated because they are guided by the haze density estimate obtained by invoking the HDE on the input image. Therefore, image blending can assign appropriate percentages to the two source images (the input image and its dehazed version) for efficient mixing. In turn, tone remapping enhances the luminance and emphasizes the color proportionately to produce a satisfactory result. Hence, the proposed AUDS can ensure superior dehazing performance in any haze condition, as verified by the results of a comparative evaluation with state-of-the-art benchmark methods. This superiority demonstrates the great potential of the proposed AUDS in facilitating other high-level vision applications, and benefiting practical systems, such as autonomous vehicles and surveillance cameras.

Although the proposed AUDS successfully processed haze-free images as well as hazy images degraded by different haze conditions, the incorrect classification of input images by the HDE during qualitative comparison indicates a potential weakness. For example, a large and homogeneous background, such as the sky or sea, exhibits characteristics similar to haze, deceiving the HDE into incorrect classification. Consequently, the proposed AUDS, whose operation depends on the HDE, fails to handle this type of image properly. This problem requires further improvement in the HDE. In particular, the HDE can be utilized together with other haze density evaluators (for example, the FADE) in a bootstrap aggregating manner to improve the classification accuracy. However, this challenging problem is a subject for further research.

## Figures and Tables

**Table 1:** Summary of real and synthetic datasets employed in this study. NA stands for not available.

Dataset	Type	Hazy Images (#)	Haze-Free Images (#)	Ground Truth
IVC	Real	25	NA	No
FRIDA2	Synthetic	264	66	Yes
D-HAZY	Synthetic	1472	1472	Yes
O-HAZE	Real	45	45	Yes
I-HAZE	Real	30	30	Yes
FINEDUST	Real	30	NA	No
500IMG	Real	NA	500	No
Dense-Haze	Real	55	55	Yes

**Table 2:** Summary of benchmark algorithms utilized in this study.

Proposed by	Abbreviation	Description
Tarel and Hautiere [16]	FFD	Fast filtering operation
He et al. [4]	DCP	Dark channel prior
Ngo et al. [39]	sIFD	Single-scale image fusion
Zhu et al. [11]	CAP	Color attenuation prior
Berman et al. [26]	NLD	Non-local haze-line prior
Cho et al. [25]	MBD	Multi-band decomposition
Cai et al. [27]	DehazeNet	Convolutional neural network
Ren et al. [46]	MSCNN	Multiscale convolutional neural network

**Table 3:** Employed parameters and their corresponding empirical values.

Component	Parameters	Empirical Values	Description
Multiscale-fusion-based dehazing	*K*	8	Number of artificially under-exposed images
	$\left\{\nu_{1},\nu_{2},\kappa_{1},\kappa_{2}\right\}$	{0.001,0.01,2.5,1}	Scaling factor generation in sharpness enhancement
	$\{\gamma_{1},\gamma_{2},\gamma_{3},\gamma_{4},$	{1,1.13,1.2,1.3,	Gamma values in artificial under-exposure
	$\gamma_{5},\gamma_{6},\gamma_{7},\gamma_{8}\}$	1.5,2.83,2.93,3}	
	*N*	Equation (4)	Number of scales in multiscale fusion
	$\Omega_{MFD}$	3×3	Patch size for weight map calculation
Haziness degree evaluator	λ	−1	Regularization coefficient
	$\gamma_{HDE}$	1/9	Emphasis strength for intensity emphasis
	$\Omega_{HDE}$	5×5	Patch size
Image blending and tone remapping	$\left\{\rho_{1},\rho_{2}\right\}$	{0.8811,0.9344}	Thresholds for range mapping
	α	0.2	Coefficient controlling range mapping curve
	θ	0.4	Coefficient controlling weight generation curve

**Table 4:** Average quantitative results on different datasets. Top three results are boldfaced in red, green, and blue.

	Method	FFD		DCP		sIFD		CAP		NLD		MBD		DehazeNet		MSCNN		AUDS	
Dataset		FSIMc	TMQI	FSIMc	TMQI	FSIMc	TMQI	FSIMc	TMQI	FSIMc	TMQI	FSIMc	TMQI	FSIMc	TMQI	FSIMc	TMQI	FSIMc	TMQI
FRIDA2	Hazy	0.7807	0.7314	0.7746	0.7291	0.7995	0.7227	0.7918	0.7385	0.7323	0.7727	0.6792	0.6512	0.7963	0.7336	0.8009	0.7232	0.7988	0.7263
	Haze-free	0.8566	0.9329	0.9586	0.9680	0.9574	0.9912	0.9102	0.8832	0.8770	0.9502	0.6668	0.5003	0.9703	0.8716	0.9656	0.9024	0.9971	0.9202
D-HAZY	Hazy	0.8703	0.8000	0.9002	0.8631	0.8640	0.7775	0.8880	0.8206	0.8395	0.8435	0.8316	0.7946	0.8874	0.7966	0.8822	0.8023	0.8685	0.7710
	Haze-free	0.8672	0.8877	0.9541	0.9123	0.9518	0.9131	0.8968	0.8829	0.8681	0.9078	0.8281	0.8758	0.9843	0.9073	0.9497	0.9075	0.9941	0.9097
O-HAZE	Hazy	0.7733	0.8416	0.8423	0.8403	0.8350	0.8991	0.7738	0.8118	0.8605	0.8915	0.8504	0.8605	0.7865	0.8413	0.8553	0.8737	0.8530	0.8983
	Haze-free	0.8379	0.8172	0.9645	0.8765	0.9192	0.8337	0.8679	0.7906	0.8253	0.8134	0.8158	0.8088	0.9839	0.8562	0.9369	0.8513	1.0000	0.9324
I-HAZE	Hazy	0.8055	0.7740	0.8208	0.7319	0.8583	0.8077	0.8252	0.7512	0.8823	0.8326	0.8607	0.8161	0.8482	0.7598	0.8631	0.7819	0.8786	0.8200
	Haze-free	0.8283	0.8380	0.9335	0.8106	0.9555	0.8911	0.8716	0.7681	0.8608	0.8565	0.8324	0.8466	0.9751	0.8343	0.9724	0.8543	0.9983	0.8979
Dense-Haze	Hazy	0.5598	0.5627	0.6419	0.6383	0.5628	0.5886	0.5773	0.5995	0.7169	0.7108	0.6867	0.6843	0.5573	0.5723	0.6029	0.6176	0.6148	0.6438
	Haze-free	0.8571	0.8440	0.9414	0.8611	0.9455	0.8771	0.8508	0.7742	0.8339	0.8346	0.8237	0.8398	0.9776	0.8539	0.9693	0.8632	0.9973	0.9171
500IMG	Haze-free	0.8645	0.8138	0.9563	0.8858	0.9366	0.8488	0.8795	0.8438	0.8855	0.8523	0.8605	0.8337	0.9870	0.8775	0.9383	0.8605	0.9990	0.8971
Total	Hazy	0.7573	0.7294	0.7746	**0.7357**	**0.7799**	0.7326	0.7693	0.7336	0.7608	**0.7856**	0.7228	0.6979	0.7725	0.7312	**0.7896**	0.7341	**0.7900**	**0.7432**
	Haze-free	0.8621	0.8293	**0.9548**	**0.8802**	0.9399	0.8597	0.8798	0.8297	0.8764	0.8564	0.8378	0.8063	**0.9840**	**0.8730**	0.9449	0.8652	**0.9986**	**0.9034**
	Overall	0.8170	0.7863	**0.8886**	**0.8272**	0.8812	0.8131	0.8392	0.7944	0.8340	**0.8304**	0.7964	0.7665	**0.9063**	0.8209	0.8879	0.8171	**0.9220**	**0.8446**

**Table 5:** Run-time in seconds on different image resolutions.

	Resolution	640×480	800×600	1024×768	1920×1080	4096×2160
Method						
FFD		0.28	0.59	0.76	1.51	9.02
DCP		12.64	19.94	32.37	94.25	470.21
sIFD		0.26	0.39	0.64	1.68	7.18
CAP		0.22	0.34	0.55	1.51	6.39
NLD		2.65	5.54	6.61	5.74	34.39
MBD		0.51	0.66	1.24	3.60	11.62
DehazeNet		1.53	2.39	3.88	10.68	47.35
MSCNN		0.54	0.88	1.53	3.43	17.90
AUDS		0.65	1.12	1.88	4.94	20.36

## Data Availability

Data available in a publicly accessible repository. The data presented in this study are openly available in [37,40,41,42,43,44] and FigShare at 10.6084/m9.figshare.14729001.v1 and 10.6084/m9.figshare.14729052.v1.
